# Adverse Respiratory Health and Hematological Alterations among Agricultural Workers Occupationally Exposed to Organophosphate Pesticides: A Cross-Sectional Study in North India

**DOI:** 10.1371/journal.pone.0069755

**Published:** 2013-07-25

**Authors:** Mohd. Fareed, Manoj Kumar Pathak, Vipin Bihari, Ritul Kamal, Anup Kumar Srivastava, Chandrasekharan Nair Kesavachandran

**Affiliations:** Epidemiology Division, CSIR - Indian Institute of Toxicology Research (CSIR-IITR), Lucknow, India; University of Rochester Medical Center, United States of America

## Abstract

**Background:**

Non-protective work practices followed by farm workers during spraying of pesticides lead to occupational exposure among them.

**Objective:**

This study is designed to explore the respiratory health and hematological profile of agricultural workers occupationally exposed to OP pesticides.

**Materials and Methods:**

A cross sectional study was undertaken among 166 pesticide sprayers working in mango orchards of Lucknow district in North India compared with 77 controls to assess the respiratory illness, lung functions, cholinesterase levels and hematological profile. A questionnaire based survey and clinical examination for respiratory health were conducted among study subjects. Lung function test was conducted among study subjects by using spirometer. Cholinesterase level as biomarker of OP pesticides and hematological profile of study subjects were investigated in the laboratory by following the standard protocols.

**Results:**

Overall respiratory morbidity observed among exposed subjects was 36.75%. Symptoms for respiratory illness like dry cough, productive cough, wheezing, irritation of throat and blood stained sputum were found to be significantly more (p<0.05) among pesticide sprayers than controls. Lung function parameters *viz.* PEFR, FEV_1_, %PEFR predicted, %FEV_1_ predicted and FEV_1_/FVC were found to be significantly decreased (p<0.05) among pesticide sprayers as compared to controls. Exposure wise distribution of respiratory illness and lung functions among pesticide sprayers show that the exposure duration significantly elevates (p<0.05) the respiratory problems and significantly decreases (p<0.001) lung functions among pesticide sprayers. Activities of acetylcholinesterase and butyrylcholinesterase were found to be significantly depleted (p<0.001) among pesticide sprayers as compared to controls which show the exposure of OP pesticides among them. The hematological profile viz. RBC, WBC, monocytes, neutrophils, MCV, MCH, MCHC and platelet count were significantly altered (p<0.001) in pesticide sprayers than controls.

**Conclusion:**

This study shows that the unsafe occupational exposure of OP pesticides causes respiratory illness, decreased lung functions and hematological alterations among pesticide sprayers.

## Introduction

In developing countries including India, agricultural workers who are engaged in the occupation of spraying pesticides in crops get the direct exposure of pesticides due to unsafe and non-preventive work practices. They do not use the Personal Protective Equipments (PPE) like safety masks, gloves etc. during the aerial spraying of pesticides resulting in the entry of pesticides in the blood stream via respiratory tract through inhalation which can adversely affect respiratory system and can also cause hematological alterations among agricultural workers.

Several earlier studies [1]–[4] have reported increased risk of respiratory problems, such as asthma, wheeze and chronic bronchitis among agricultural workers. Pesticides after reaching to the lungs from the systemic circulation through inhalation and absorption, adversely affect the lung tissues [5]. The health effects of pesticide inhalation through occupational exposure have been documented in a number of epidemiologic studies reporting higher risk of respiratory symptoms [4], [6], [7]. Apart from respiratory symptomatology, a relatively small number of studies have attempted to assess the pulmonary function (by means of spirometry) after long-term pesticide exposure. Some earlier studies reported the higher prevalence of respiratory symptoms supported by reduced lung function test among agricultural workers occupationally exposed to pesticides [7]–[8].

Pesticides have been shown to have hematotoxic properties and may cause aplastic anemia, agranulocytosis, neutropenia, and thrombopenia [9]. Both acute and chronic exposure to toxic doses of pesticides may induce hematological, congenital abnormalities and thalassemia [10]. More interaction of organophosphate pesticides (OP) with iron results in lesser binding efficiency of Hemoglobin with iron leading to anaemic conditions [11].

OP pesticides used by the agricultural workers inhibit the action of AChE, enzyme involved in the release of acetylcholine at the nerve endings [12], thus OP increases the cholinergic effects of acetylcholine in the body and depolarization of neural transmission [13]. Acetylcholinesterase (AChE) activity in the red blood cells and Butrylcholinesterase (BChE) activity in plasma have been used to monitor the extent of exposure of organophosphates (OP) which are considered as cholinesterase inhibiting pesticides [12]–[13]. There is a correlation between exposure to OP pesticides and inhibition of AChE activity [14]–[15]. Therefore, the level of AChE activity has been considered a reliable biomarker for exposure to these pesticides [15]–[18].

The studies from Asian sub-continent including India is very important keeping in view of the incorrect work practices, non-usage of PPE etc. practised by pesticide sprayers during spraying operations in the field. Literature on the association of pesticide exposure with adverse respiratory health and hematological alterations among agrarian communities of Asian countries are scanty. In the present study we investigated the altered hematological profile along with symptoms of respiratory illness, lung function tests FEV_1_ (Forced Expiratory Volume in 1 second), PEFR (Peak Expiratory Flow Rate) and FVC (Forced Vital Capacity) for pulmonary impairment among agricultural workers in rural agricultural sector of Lucknow district in India. These subjects spray OP pesticides during agricultural work practices in unsafe environment without following any preventive and hygienic work practices. Cholinesterase levels as a biomarker of OP pesticides was also investigated to ascertain the exposure of OP pesticides among agricultural workers. The present study aims to explore the respiratory health and hematological profile among agricultural workers occupationally exposed to OP pesticides in North India.

## Materials and Methods

### Study Design

**Figure 1:**
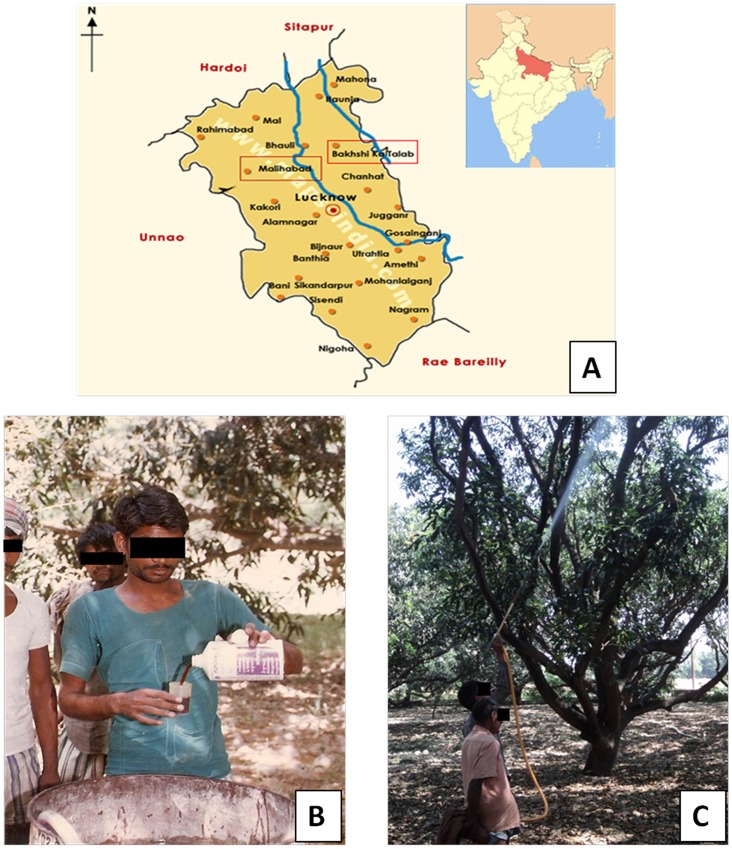
Study location and pesticide exposed subjects. (A) Map showing Malihabad and Bakshi Ka Talab regions of Lucknow district, figure in inset is map of India showing Uttar Pradesh state (dark filled); (B) Figure showing the handling of pesticide with bare hands by a sprayer; (C) Figure showing the spraying of pesticides without personal protective devices by sprayers.

This is a cross sectional study for respiratory health related to agricultural pesticide exposure. The study was conducted at Malihabad and Bakshi Ka Talab regions of Lucknow district located in North India (Figure 1A). These areas have several mango plantations where agricultural workers spray pesticides to protect the mango crop from harmful pests and thereby enhancing the mango crop productivity. The pesticide sprayers have experience of spraying pesticides in mango plantation from 1–30 years. Spraying operations last for 2 to 8 hour/week per season on the trees of mango orchards from December to March every year. Mixing of chemicals with bare hands, leakages from tanks of pesticide during spraying operations were found to be very common. Sprayers of mango plantations were using OP pesticides like monocrotphos, dichlorvos, malathion, methyl parathion etc.

### Subjects

We selected subjects on the basis of the results of a preliminary questionnaire specifically designed for this study. All the subjects were engaged in the spraying of OP pesticides without using any PPE. One hundred sixty six male pesticide sprayers participated in the study. The study was compared with 77 healthy males having no previous or current occupational exposure to pesticides taken as control from near by area with same socio-economic status. On the basis of area of living, ethnicity of both groups of subjects was almost similar. The purpose of the study was explained to all the participants and their consent was obtained. Subjects of the photograph shown in Figure 1(B) and 1(C) have given written informed consent, as outlined in the PLOS consent form, to publication of their photograph. All the health surveys, spirometry and collection of blood samples of pesticide sprayers were performed during the end phase of the pesticide application season in the month of March with time interval of 1–2 days after the last spraying operation.

Pesticide sprayers were randomly selected on the basis of inclusion and exclusion criteria.

Inclusion criteria of pesticide sprayers (exposed subjects) include:

1. Male pesticide sprayers with age group of 18–60 yrs.
2. Pesticide sprayers must have at least six months previous experience in spraying operations in the field.

Exclusion criteria of pesticide sprayers (exposed subjects) include:

1. Pesticide sprayers less than 18 yrs of age and greater than 60 yrs.
2. Pesticide sprayers with less than six months work experience.
3. Female subjects.

Control subjects were randomly selected on the basis of inclusion and exclusion criteria.

Inclusion criteria of control subjects include:

Male resident who did not handle pesticides and having similar socio economic status and age group of exposed subjects.

Exclusion criteria of control subjects include:

1. Male subjects with age group less than 18 yrs of age and greater than 60 yrs and those handling pesticides.
2. Female subjects

The criteria for the withdrawal of subjects: If sprayer or control subject did not wish to participate in the study after giving detail explanation of the study, he was free to discontinue/withdraw at any stage of procedure *viz.,* collection of personal background information or during clinical procedures.

### Questionnaire

A preliminary questionnaire was designed to record the personal and occupational information along with pesticide exposure and respiratory health status of the pesticide sprayers. Each pesticide sprayer was interviewed based on questionnaire like general information about the name, age, smoking habit of individual pesticide sprayer; information about the exposure period of pesticide spraying in respect of years; information about the use of pesticide like the type of pesticide they spray, methods of spraying the pesticides, information about the work practices followed by them during spraying the pesticides such as whether they handle the pesticides with bare hands or measuring cup, whether they wear any protective equipment, whether they eat anything while spraying, whether they take bath after spraying etc.; respiratory health information through clinical examination.

### Clinical Examination

Clinical examination for respiratory health was done by a medical expert and the symptoms for respiratory illness were recorded in the questionnaire for every individual who participated in this study. Human ethical clearance was obtained from “Human Ethics Committee” of Indian Institute of Toxicology Research for collecting the blood samples and clinical investigations of the participants. No minors/children participants were involved in this study. All the participants were above the age of 18 years. All the participants provided their written informed consent to participate in this study which was approved by Institutional Human Ethics Committee. All the documents including questionnaires and consent forms of all the participants were safely kept for data analysis.

### Blood Sample Collection

Approximately 3.0 ml venous blood was collected in the heparinised glass vials as coded samples from both the control and sprayers. Samples were transported to the laboratory of Epidemiology division of Indian Institute of Toxicology Research, Lucknow, India in ice-cold condition immediately after the collection. The hematological investigation and cholinesterase measurement were carried out on same day of blood collection.

### Chemicals and Reagents

5,5′-dithio-bis-(2-nitrobenzoic acid) (DTNB), sodium dodecyl sulphate (SDS), tris HCl buffer, acetylthiocholine iodide and butrylthiocholine iodide, were from Sigma Chemical Co., St. Louis, MO. All the other chemicals used were of the highest purity available from commercial sources.

### Cholinesterase Measurement

Blood AChE and BChE activities were assayed by the method Ellman et al, 1961 [19] as modified by Chambers and Chambers, 1989 [20] by taking acetylthiocholine iodide and butyrylthiocholine iodide respectively as substrate and expressed as mmols hydrolyzed/h/l blood (IU/L). The AChE and BChE were measured as a biomarker of OP pesticides exposure among study subjects.

### Hematology

Standard protocol for hematological investigation was undertaken on each sprayer and controls. Hematological indices studied were total red blood count, total leukocyte count, differential leukocyte count, hemoglobin percentage, hematocrit, mean corpuscular volume (MCV), mean corpuscules hemoglobin concentration (MCH) and platelet count. Hematology analyzer (Arcus, Diatron) was used for complete blood count. The procedure followed was as per the instruction manual of Hematology Analyser (Arcus, Diatron). 25 µl of anti-coagulated whole blood sample was aspirated by the sampling needle (present in sampling bar), and 4 ml of diluent was added into the chamber. 25 µl of primary dilution was aspirated and stored in the needle during the WBC measurement. 0.9 ml of lysing reagent was added to the primary dilution remaining in the chamber. After the WBC measurement and a washing process, the instrument made the second dilution into the chamber with the sample stored in the needle and with 5 ml of diluent reagent, then the other hematological parameters were detected. The lysing agent and diluent were provided with the instrument. Both the lower limit and upper limit from the normal range for each hematological parameter was taken as cases with variation.

### Lung Function Test

Lung function tests FEV_1_ (Forced Expiratory Volume in 1 second), PEFR (Peak Expiratory Flow Rate) and FVC (Forced Vital Capacity) for pulmonary impairment of each subject (pesticide sprayers and control) were performed using a Spirometer (PIKO-1, UK under the recommendation of the American Thoracic Society standards). The purpose of the lung function test was to evaluate pulmonary impairments among study subjects. The volunteer performed the lung function test three times allowing for sufficient rest between repetitions. The best values for PEFR, FEV_1_ and FVC from three tests for each subject were recorded. Results were interpreted with the predicted values of lung function parameters calculated by reference equation for Indian population [21].

### Statistical Analysis

Descriptive statistics have been generated for the demographic parameters in the control group and pesticide sprayers. Frequencies and percentages have been shown for all the categorical parameters. Student’s t test has been used to compare the mean values of the quantitative characteristics (demographic parameters, spirometry parameters and cholinesterase activity) between the control and exposed group. Chi square test has been incorporated for comparison of the categorical outcomes (hematological abnormalities, pulmonary impairments, respiratory problems). Fisher’s exact test has been used in cases where the expected cell frequencies were less than 5. Chi-square test for linear trend was used for exposure wise distribution of respiratory illness among pesticide sprayers. The calculation of odds ratio with 95% confidence interval (CI) for respiratory and hematological alterations in respect to potential risk factors has been performed using logistic regression models. Odds ratio for respiratory symptoms were adjusted for smoking using multivariate logistic regression analysis. Linear regression analysis was done considering PEFR and spray duration as independent variables and FEV_1_ and PEFR as the dependent variables respectively. The criterion for significance was set at *p*<0.05. All the statistical analysis has been performed using EPI INFO 7.1.1.0 software (Centre for Disease Control and Prevention, Georgia, USA) and IBM SPSS Statistics version 20.

## Results

### Physical Characteristics, Personal Habits and Exposure Period of Study Subjects

Table 1 exhibits the physical characteristics, personal habits and exposure period of study subjects. Age, height, weight and BMI were found to be almost similar among controls and pesticide sprayers. Smokers and alcohol consumers were found to be insignificantly more in pesticide sprayers group than controls; whereas tobacco chewers were found to be insignificantly more in controls group than pesticide sprayers group. Mean pesticide exposure among sprayers was found to be 17.91±11.68 years.

**Table 1:** Physical characteristic, personal habits and exposure period of study subjects.

Physical characteristics and personal habits	Controls (N = 77)	Pesticide sprayers (N = 166)
Age (yrs) [Mean ± SD]	37.28±14.13	38.12±15.39
Weight (Kg) [Mean ± SD]	52.76±13.54	51.32±7.56
Height (cm) [Mean ± SD]	164.24±7.53	163.94±6.16
BMI (Kg/m^2^)	20.03±4.34	19.08±2.48
Smokers [n (%)]	26 (33.76)	62 (37.34)
Tobacco chewers [n (%)]	23 (29.87)	41 (24.69)
Alcohol consumers [n (%)]	11(14.28)	39 (23.49)
Pesticide exposure (yr.)	0.0±0.0	17.91±11.68**

** p<0.01.

### Information about the Agricultural Work Practices followed by Pesticide Sprayers

Table 2 shows the information about different agricultural work practices followed by pesticide sprayers which are based on preliminary questionnaire designed for this study. Information about the place of pesticide storage, place for pesticide preparation and handling, personal protecting equipment and awareness during mixing/loading/spraying, fate of empty packages/containers of pesticide are shown in this table.

**Table 2:** Information about the agricultural work practices followed by pesticide sprayers.

Variables	Pesticide sprayers (N = 166) n (%)
**Place for pesticide storage**	
Inside the house	73 (43.98)
Inside plantation areas	20 (12.05)
No storage	30 (18.07)
Tools storage room	50 (30.12)
**Place for pesticide preparation and handling**	
In home	30 (18.07)
In fields	80 (48.19)
Near pond	70 (42.17)
Handling of pesticide container by bare hands	166 (100)
Mixing of pesticide by bare hands	60 (36.14)
Mixing of pesticide by rod	20 (12.05)
Mixing by motor	100 (60.24)
**Personal protecting equipment and awareness during mixing/loading/spraying**	
Use of gloves/goggles/apron	0 (0)
Cloths wet during spraying	120 (72.29)
Smoke while spraying	45 (27.11)
Eat in the break between spraying	75 (45.18)
Take bath after the end of spraying	140 (84.34)
Change clothes at the end of work shift	135 (81.32)
Wash contaminated clothes with family clothes	115 (69.28)
Any integrated pest management training taken	0 (0)
**Fate of empty packages/containers of pesticide**	
Buried/burned	0 (0)
Discarded near the river/canal/field	113 (68.07)
Use at home	35 (21.08)
Sold to scrap dealer	17 (10.24)
Used for some other work	15 (9.04)

### Self Reported Respiratory Problems among Study Subjects

Table 3 shows various respiratory problems *viz.* dry cough, productive cough, dyspnoea, wheezing, irritation of throat, blood stained sputum, observed among pesticide sprayers and control subjects stratified by smoking habits. Prevalence of overall respiratory problems among study subjects which was found to be 36.75% (AOR- 14.33; 95% CI- 4.37–73.52) which was highly significant (p<0.01) as compared to controls (3.89%); respiratory problems like dry cough (AOR- 10.41; 95% CI- 1.59–437.09), Dyspnoea (AOR- 2.15; 95% CI- 0.43–20.87) were found to be significantly more among pesticide sprayers as compared to controls. No cases among control group were observed for productive cough, wheezing, irritation of throat and blood stained sputum.

**Table 3:** Respiratory symptoms observed among study subjects.

Respiratory problems	Controls (N = 77) n (%)			Pesticide sprayers (N = 166) n (%)			Adjusted odds ratio#	95% CI
	Smokers	NonSmokers	Total	Smokers	NonSmokers	Total		
Prevalence of overall respiratory problems	2 (2.59)	1 (1.29)	3 (3.89)	26 (15.66)	35 (21.08)	61 (36.75)**	14.33	4.37–73.52
Dry cough	1 (1.29)	0 (0.0)	1 (1.29)	8 (4.81)	12 (7.22)	20 (12.05)**	10.41	1.59–437.09
Productive cough	0 (0.0)	0 (0.0)	0 (0.0)	12 (7.22)	19 (9.63)	31 (23.49)**	–	–
Dyspnoea	1 (1.29)	1 (1.29)	2 (2.59)	3 (1.80)	6 (3.61)	9 (5.42)**	2.15	0.43–20.87
Wheezing	0 (0.0)	0 (0.0)	0 (0.0)	4 (2.40)	5 (3.01)	9 (5.42)**	–	–
Irritation of throat	0 (0.0)	0 (0.0)	0 (0.0)	1 (0.60)	2 (1.20)	3 (1.81)**	–	–
Blood stained sputum	0 (0.0)	0 (0.0)	0 (0.0)	2 (1.20)	1 (0.60)	3 (1.81)**	–	–

** p<0.01;

# Odds ratio adjusted for smoking habits.

### Lung Function Tests among Study Subjects

Table 4 exhibits lung function parameters *viz.* PEFR, FEV_1,_ %PEFR predicted, %FEV_1_ predicted, FVC, FEV_1_/FVC of controls and pesticide sprayers group in which all the parameters for lung functions except FVC were found to be significantly decreased (p<0.01) among pesticide sprayers as compared to controls.

**Table 4:** Lung function tests among study subjects.

Lung function parameters	Controls N = 77)	Pesticide sprayers (N = 166)
PEFR (Lt/min) [Mean ± SD]	440.44±112.45	306.34±108.46**
FEV _1_ (Lt) [Mean ± SD]	2.26±0.59	1.76±0.63**
%PEFR predicted [Mean ± SD]	81.09±20.34	60.03±20.74**
%FEV1 predicted [Mean ± SD]	77.95±18.17	64.41±24.73**
FVC (Lt) [Mean ± SD]	2.23±0.74	2.20±0.77
FEV1/FVC [Mean ± SD]	0.84±0.01	0.79±0.01**

** p<0.01.

### Exposure Wise Distribution of Respiratory Illness

Table 5 shows exposure wise distribution of respiratory illness among pesticide sprayers which illustrates that exposure time elevates the respiratory problems among pesticide sprayers. Pesticide exposure was taken into consideration as product of number of years and number of hours (no. of years×no. of hours); number of hours are the average hours of spraying pesticides per week throughout the year, and according to which 4 groups were established, namely <11, 11–50, 51–100, 101 and above. It was seen that the group having most time of pesticide exposure exhibited most prevalence of respiratory problems. Odd ratio also shows regular increment pattern as exposure time increases, chi-square value for linear trial was found to be 5.09 and p-value was found to be 0.02 which shows statistical significance for increasing trend in prevalence of respiratory illness observed with increasing pesticide exposure.

**Table 5:** Exposure wise distribution of respiratory illness among pesticide sprayers.

Spraying duration(no. of years×no. of hours)	No. of subjects (%)	No. of cases havingrespiratory illness (%)	Odds ratio*
<11	60 (36.14)	16 (26.67)	1.00
11–50	54 (32.53)	20 (37.03)	1.62
51–100	32 (19.28)	15 (46.87)	2.43
101 and above	20 (12.05)	10 (50.00)	2.75

* Chi square for linear trend is 5.087 (p-value = 0.02).

### Activities of Cholinesterase Enzymes

The biomarkers of organophosphate pesticides exposure *viz.*, AChE and BChE activity in blood among pesticide sprayers was analysed and the results shown in Table 6. Activities of both AChE and BChE among pesticide sprayers were found to be significantly depleted (p<0.01) as compared to control subjects.

**Table 6:** Cholinesterase activity (IU/L) among study subjects.

Cholinesterase activity (IU/L)	Control (n = 77)	Pesticide sprayers (n = 166)
Acetylcholinesterase [Mean ± SD]	570.24±306.71	420.28±239.62**
Butyrylcholinesterase [Mean ± SD]	434.91±218.46	253.76±186.30**

** p<0.01.

### Hematological Findings

Frequency distribution of hematological variations among study subjects are shown in Table 7. Significant (p<0.05) abnormal counts considering both below lower limit and above upper limit from normal range were found for R.B.C., W.B.C., Neutrophils, Hemoglobin, MCV, MCHC and platelets among pesticides sprayers as compared to control subjects. Table 8 shows the association of respiratory problems with hematology among study subjects showing significantly (p<0.01) increasing hematological alterations (OR- 3.94; 95% CI- 2.02–7.67) among subjects with respiratory problems as compared to those subjects without respiratory problems.

**Table 7:** Frequency distribution of abnormal hematological profile among study subjects.

Hematological Profile	Normal values of hematology	Control subjects with abnormal hematology (N = 77) n (%)			Pesticide sprayers with abnormal hematology (N = 166) n (%)		
		Below lower limit	Above upper limit	Total subjects	Below lower limit	Above upper limit	Total subjects
R.B.C.	4.0–5.5 (X 10^6 ^mm^−3^)	5 (6.49)	4 (5.19)	9 (11.69)	32 (19.28)	26 (15.66)	58 (34.94) **
W.B.C.	5.00–11.00 (X 10^3^ mm^−3^)	1 (1.29)	2 (2.59)	3 (3.89)	14 (8.43)	22 (13.25)	36 (21.69) **
Lymphocytes	20–50 (%)	5 (6.49)	6 (7.79)	11 (14.28)	26 (15.66)	8 (4.82)	34 (20.48)
Monocytes	2.0–12.0 (%)	0 (0.0)	3 (3.89)	3 (3.89)	33 (19.88)	32 (19.28)	65 (39.16) **
Neutrophils	35–80 (%)	0 (0.0)	1 (1.29)	1 (1.29)	30 (18.07)	8 (4.81)	38 (22.89) **
Hemoglobin	12.0–17.4 (g/dl)	5 (6.49)	0 (0.0)	5 (6.49)	28 (16.87)	3 (1.81)	31 (18.67) *
MCV	76–96 (femtoliters)	11 (14.28)	7 (9.09)	18 (23.38)	32 (19.28)	24 (14.46)	56 (33.73) *
MCH	27–32 (pg)	15 (19.48)	10 (12.98)	25 (32.47)	39 (23.49)	28 (16.87)	67 (40.36)
MCHC	30–35 (%)	18 (23.38)	9 (11.69)	27 (35.06)	52 (31.32)	31 (18.67)	83 (50.0) *
Platelet count	150–450 (X 10^3^ mm^−3^)	1 (1.29)	1 (1.29)	1 (1.29)	42 (25.30)	5 (3.01)	47 (28.31) **

* p<0.05; ** p<0.01; R.B.C.- Red Blood Cells, W.B.C.- White Blood Cells, MCV- Mean Corpuscular Volume, MCH- Mean Corpuscular Hemoglobin, MCHC- Mean Corpuscular Hemoglobin Concentration.

**Table 8:** Association of respiratory problems with hematology among pesticide sprayers.

Subjects	With respiratory symptoms		Without respiratory symptoms	
	Having normalhaematology	Having alteredhaematology*	Having normalhaematology	Having alteredhaematology*
Pesticide sprayers (n = 166) n (%)	23 (13.85)	38 (22.90)	74 (44.60)	31 (18.68)
Controls (N = 77) n (%)	1 (1.2)	2 (2.59)	58 (75.32)	16(20.77)

* Altered haematology is considered if one or more than one parameters are out of normal range.

Odds Ratio = 3.94 (95% CI = 2.02–7.67); p<0.01.

### Correlations of Lung Function Parameters with Cholinesterase and Spray Duration

Correlations of all the lung function parameters *viz.* PEFR, FEV_1,_ %PEFR predicted, %FEV_1_ predicted, FVC, FEV_1_/FVC with AChE, BChE and spray duration among pesticide sprayers are shown in Table 9. PEFR is significantly correlated (p<0.01) with spray duration; FEV_1_ is significantly correlated (p<0.01) with AChE and spray duration; %PEFR predicted is significantly correlated (p<0.01) with spray duration; predicted %FEV_1_ predicted is significantly correlated (p<0.01) with AChE; FVC is significantly correlated (p<0.01) with AChE and spray duration; FEV_1_/FVC is significantly correlated (p<0.01) with AChE and spray duration.

**Table 9:** Linear regression analysis of Lung function parameters with AChE, BChE and spray duration among pesticide sprayers.

Riskfactors	Statisticalcoefficients	AChE	BChE	Sprayduration
PEFR	r value	−0.17	0.06	−0.41
	β coefficient	−0.11	0.01	−0.41
	F value	−1.19	0.16	−4.75
	*p* value	0.24	0.88	<0.01
FEV_1_	r value	−0.26	0.05	−0.37
	β coefficient	−0.24	0.07	−0.30
	F value	−2.60	0.78	−3.48
	*p* value	<0.05	0.43	<0.01
%PEFRpredicted	r value	−0.15	0.03	−0.27
	β coefficient	−0.11	0.02	−0.26
	F value	0.17	−1.15	−2.86
	*p* value	0.87	0.25	<0.01
%FEV_1_predicted	r value	−0.19	−0.06	0.01
	β coefficient	−0.22	0.04	0.07
	F value	0.39	−2.21	0.73
	*p* value	0.69	<0.05	0.47
FVC	r value	−0.26	0.04	−0.35
	β coefficient	−0.24	0.07	−0.29
	F value	−2.58	0.76	−3.32
	*p* value	0.45	<0.05	<0.01
FEV_1_/FVC	r value	−0.20	0.12	−0.59
	β coefficient	−0.13	0.06	−0.56
	F value	−1.58	0.71	−7.41
	*p* value	0.48	0.115	<0.01

## Discussion

Pesticide sprayers in the present study were unaware of the health risks associated with spraying, as most of them were illiterate and did not used any precautionary measures while spraying of pesticides. These pesticide sprayers never used PPE during spraying operations. Mixing of chemicals with bare hands and pesticide leakage from tanks during spraying operations was found to be very common. Due to upward spraying in the mango orchards even with mechanized spraying equipment, pesticides fell onto the body of the sprayers. During spraying, these workers wore minimum clothes, *i.e*. only underwear and not even vests because of the tropical climate in North India.

Inhalation of pesticides on the worksite occurs, provided that one or more of the following conditions exist: the chemical in use is quite volatile; application is in an enclosed poorly ventilated area, or the manner of application leads to an aerosol cloud of finely dispersed droplets that do not readily settle [22]. Small aerosol droplets of pesticides during spraying remain suspended in the air and may be inhaled by pesticide sprayers. Once inhaled, pesticide aerosols or vapors may react with airways, producing irritant effects and further airway narrowing. Moreover, if the droplet size is small enough it can reach the alveolar space and damage the alveolar capillary membrane, leading to gas diffusion impairment [23].

In the present study, respiratory symptoms like dry cough, productive cough, wheezing, irritation of throat and blood stained sputum were found to be significantly more among pesticide sprayers. Parameters of lung function tests *viz.* PEFR, FEV_1,_ %PEFR predicted, %FEV_1_ predicted, FEV_1_/FVC were found to be significantly less among pesticide sprayers which show the pulmonary impairments among them. Lung function impairments were found to be more among pesticide sprayers in comparison to control subjects, which is in agreement with an earlier study [24] reporting that pesticides can cause laryngeal and bronchial constriction. We have correlated the exposure duration of pesticides in years and hours with the prevalence of respiratory symptoms and found a regular increment pattern in prevalence of respiratory symptoms which illustrates that exposure duration causes more respiratory illness due to pesticides. Likewise exposure wise distribution of PEFR, FEV_1,_ %PEFR predicted, FEV_1_/FVC among pesticide sprayers shows that exposure duration of pesticides increases the lung function alterations. Recently our group has reported significant depletion in blood AChE activity and FEV_1_ among pesticide sprayers in post spraying season as compared to pre spraying season [25]. Several epidemiological and toxicological studies have shown results comparable to those of present study. A cross sectional study was conducted in Canada on 1939 farmers for pesticides and their respiratory effects: pesticide use was associated with isolated asthma and pulmonary function change [26]. Some earlier studies depicted that pesticides associated with respiratory symptoms included organophosphates, thiocarbamates, paraquat, [27] and carbamates [26]. In the Iowa farm family health and hazard surveillance project, among farmers, a study among agricultural workers in Iowa found a clear association of pesticide use with respiratory symptoms [28]; in Ohio, rural work involving pesticides was related to the significantly increased chronic cough [3]. The observation of a decreased FEV_1_ with depleted cholinesterase levels among pesticide sprayers in present study shows agreement with some earlier studies in which they showed short-term exposure to anticholinesterase insecticides (OP pesticides) lead to transient obstructive lung dysfunction owing to large airway narrowing [23], [29], [30].

Anticholinesterase pesticides *i.e* organophosphates used by the pesticide sprayers in the present study were neurotoxic in nature, so the AChE and BChE activities were found to be significantly depleted among the pesticide sprayers than that of control group. The decreased activity of AChE and BChE in the exposed subjects can be related to OP pesticide exposure in the present study considering them as good indicators for OP toxicity. Cholinesterase depletion is well correlated with use of organophosphate pesticides during the farming season [8], [31]. In some earlier studies, AChE inhibition was found to be significantly associated with OP pesticide toxicity [32]–[33]. AChE inhibition was observed among desert farm workers exposed to OP and carbamates [34]. The depletion of AChE reported in the UAE workers occupationally exposed to OP pesticides correlated well with the frequency of usage and length of use [8], [35]–[36]. Decreased activity of BChE due to OP exposure in the present study supports the earlier report [37]. In a similar study [38], inhibition of BChE due to OP exposure was reported but results were non-significant. As in our study, similar observations were found in some earlier studies where pesticide sprayers having respiratory illness and altered lung functions showed depleted cholinesterase activities [23], [29].

The alterations observed in the hematological parameters of sprayers can be related to OP pesticides exposure which is evidenced by decreased AChE and BChE activity. RBC, WBC, monocytes, neutrophils, MCV, MCH, MCHC and platelet count of sprayers show significant alterations compared to controls which can be related to OP pesticide exposure. Similar observation on abnormal haematogical profile *viz.* haematocrit, RBC, WBC and platelet count was previously reported among pesticide exposed farm workers [39]–[40]. Excess risks of Chronic Lymphoid Leukemia and Multiple Myleloma have been found in various studies among farmers [41]–[43], and pesticide exposure has been suggested as the cause. Variations in WBC, hemoglobin, hematocrit and platelets count was also reported in pesticide exposed population in a study with small sample size [37]. Hematological abnormalities including instances of leukopenia, leucocytosis, lymphocytopenia, lymphocytosis, neutropenia, monocytosis, anaemia and thrombocytopenia were reported in the exposed subjects in the present study. Leukocytosis with abnormal count of lymphocytes and monocytes due to pesticide exposure have been reported in earlier human studies [44]–[45]. The decrease in RBC count and hemoglobin content may be due to disruptive action of the pesticides on the erythropoietic tissue as a result of which the viability of the cells might be affected [46]. In an earlier report [47], aplastic anaemia was found among farm workers exposed to agricultural pesticides. Hematological alterations observed in this study could be due to exposure of OP pesticides or due to respiratory problems observed among the pesticide sprayers.

### Conclusion

It is concluded from this study that agricultural workers who are occupationally exposed to OP pesticides, showed adverse respiratory health and hematological alterations. The exposure of OP pesticides among them caused various respiratory problems, altered lung functions and hematological abnormalities. Due to unawareness of the hazardous health effects of pesticides among agricultural workers, they followed incorrect work practices without taking any safe and precautionary measures of handling and spraying pesticides, which eventually resulted in exposure of pesticides among them. It is very necessary to monitor health risks associated with pesticide exposure regularly among agricultural workers and make awareness programme with implementation of proper legislations for safe methods of spraying pesticides, so that the adverse health effects among agricultural workers could be minimised.
