# Correction: Adverse Respiratory Health and Hematological Alterations among Agricultural Workers Occupationally Exposed to Organophosphate Pesticides: A Cross-Sectional Study in North India

**DOI:** 10.1371/annotation/b7bc0625-6200-4433-9971-f4e571203432

**Published:** 2013-08-02

**Authors:** Mohd. Fareed, Manoj Kumar Pathak, Vipin Bihari, Ritul Kamal, Anup Kumar Srivastava, Chandrasekharan Nair Kesavachandran

There was an error in the second sentence of the Funding statement. The correct second sentence is: "The publication charges have been funded by institutional project BSC-0111." 

